# The Importance of Being on Time: Regulatory Networks Controlling Photoperiodic Flowering in Cereals

**DOI:** 10.3389/fpls.2017.00665

**Published:** 2017-04-26

**Authors:** Vittoria Brambilla, Jorge Gomez-Ariza, Martina Cerise, Fabio Fornara

**Affiliations:** Department of Biosciences, University of MilanMilan, Italy

**Keywords:** photoperiod, florigen, cereals, flowering, gene regulatory network

## Abstract

Flowering is the result of the coordination between genetic information and environmental cues. Gene regulatory networks have evolved in plants in order to measure diurnal and seasonal variation of day length (or photoperiod), thus aligning the reproductive phase with the most favorable season of the year. The capacity of plants to discriminate distinct photoperiods classifies them into long and short day species, depending on the conditions that induce flowering. Plants of tropical origin and adapted to short day lengths include rice, maize, and sorghum, whereas wheat and barley were originally domesticated in the Fertile Crescent and are considered long day species. In these and other crops, day length measurement mechanisms have been artificially modified during domestication and breeding to adapt plants to novel areas, to the extent that a wide diversity of responses exists within any given species. Notwithstanding the ample natural and artificial variation of day length responses, some of the basic molecular elements governing photoperiodic flowering are widely conserved. However, as our understanding of the underlying mechanisms improves, it becomes evident that specific regulators exist in many lineages that are not shared by others, while apparently conserved components can be recruited to novel functions during evolution.

## Introduction

Several plant species measure day length to start specific developmental switches, e.g., the transition to reproductive growth, during the most appropriate times of the year. Seasonal variation of day length provides a fundamental parameter to synchronize developmental changes, because it is not subject to fluctuations like other environmental cues such as temperature.

Plants can be categorized as long day (LD) or short day (SD) species, depending on the photoperiod most effective at triggering reproductive growth. When day length exceeds a specific critical threshold, flowering is promoted in LD plants, whereas SD plants flower in response to reduction of day length below a critical threshold. Such thresholds are characteristic of each species and largely determined by the region where the species originated and first adapted. Plants growing at low tropical latitudes tend to flower in response to exposure to long nights, whereas species adapted to higher latitudes promote flowering during seasons characterized by LD, indicative of the warm days of spring and summer. Plants adapted to temperate regions that germinate before winter, often also need to satisfy a vernalization requirement (exposure to low non-freezing temperatures for several weeks) to become competent to respond to photoperiodic induction. Additionally, many plants can promote flowering even after long exposures to non-inductive photoperiodic conditions, indicating a facultative response to day length and the existence of floral promoting stimuli that can bypass the requirement for specific conditions. Therefore, plant interactions with its growth environment can be complex, and gene networks have evolved that respond to changing seasonal parameters.

In crop species, responses to day length have been extensively manipulated, creating varieties that can grow, flower and set seeds at latitudes outside of the range occupied by the wild progenitor. Artificial adaptation to broad latitudinal ranges has been a key step during domestication of several species, allowing cultivation and diversification in many regions of the globe. Natural genetic variation has offered the substrate for human selection and remarkably, many domestication loci encode orthologous genes in distantly related species providing a molecular perspective to look at conservation and evolution of pathways regulating flowering.

Here, we will summarize recent advances in understanding photoperiodic flowering regulation in crop species, focusing on cereals. Starting with the tenets established using Arabidopsis as model system, we will discuss how conserved and unique elements have been deployed to evolve flowering networks of LD and SD plants and how they control production of a florigenic systemic signal in leaves.

## Arabidopsis Contributed to Develop the Basic Tenets of Photoperiodic Flowering

Photoperiodic flowering has been mostly studied using the dicot Arabidopsis, through which core genetic and molecular mechanisms at the base of the process have been characterized (Song et al., 2015). Arabidopsis might not be fully representative of all plant species but it provides a conceptual framework that can be implemented in other species and also used to discuss evolution of distinct mechanisms typical of distantly related plants (**Table 1**).

**Table 1:** List of genes controlling photoperiodic flowering.

Arabidopsis	Rice	Maize	Sorghum	Wheat	Barley
*PhyB* (AT2G18790)	*OsPhyB* (LOC_Os03g19590)	*ZmPhyB1* (GRMZM2G124532) *ZmPhyB2* (GRMZM2G092174)	*SbPhyB* (Sb01g037340)	*TaPhyB* (AY888046)	*HvPhyB* (DQ201142)
n.p.	*Ehd1* (LOC_Os10g32600)	*ZmEhd1* (GRMZM2G479110)	*SbEhd1* (Sb01g019980)	n.f.	n.f.
n.p.	*Ehd2/RID1/OsID1* (LOC_Os10g28330)	*ID1* (GRMZM2G011357)	*SbID* (Sb01g021480)	n.f.	*HvID1* (AK361456)
n.p.	*Ghd7* (LOC_Os07g15770)	*ZmCCT* (GRMZM5G868285)	*SbGhd7* (Sb06g000570)	n.f.	n.f.
*NF-YB2* (AT5G47640) *NF-YB3* (AT4G14540)	*Ghd8* (LOC_Os08g07740)	*ZmGhd8* (GRMZM2G444073)	*SbGhd8* (Sb07g004740)	*TaNFYB-A6* (Traes_2AL_AE22E725E) *TaNFYB-B6* (Traes_2BL_3237AA694) *TaNFYB-D6* (Traes_2DL_DA577AF57)	*HvNF-YB7* (MLOC_57782)
*GI* (AT1G22770)	*OsGI* (LOC_Os01g08700)	*gigz1A/gi1* (GRMZM2G107101) *gigz1B/gi2* (GRMZM5G844173)	*SbGI* (Sb03g003650)	*TaGI1* (AF543844) *TaGI2* (AY679114) *TaGI3* (AY679115)	*HvGI* (AY740523)
*FKF1* (AT1G68050)	*OsFKF1* (LOC_Os11g34460)	*ZmFKF1a* (GRMZM2G107945) *ZmFKF1b* (GRMZM2G106363)	*SbFKF1* (Sb05g021030)	*TaFKF1* (DQ923399)	*HvFKF1* (FJ913271)
*CDF1* (At5g62430) *CDF2* (At5g39660) *CDF3* (At3g47500) *CDF5* (At1g69570)	*OsDOF12* (LOC_Os03g07360)	n.f.	n.f.	n.f.	n.f.
*PRR3* (AT5G60100) *PRR7* (AT5G02810)	*PRR37* (LOC_Os07g49460)	*ZmPRR37* (GRMZM2G033962 and GRMZM2G005732)	*SbPRR37* (Sb06g014570)	*Ppd-D1* (AB646976)	*HvPpd-H1* (AAY42109)
*CO* (AT5G15840)	*Hd1* (LOC_Os06g16370)	*conz1* (GRMZM2G405368)	*SbCO* (Sb10g10050)	*TaHd1-1* (AB094487) *TaHd1-2* (AB094488) *TaHd1-3* (AB094489)	*HvCO1* (AF490468) *HvCO2* (AF490470)
*FT* (AT1G65480)	*Hd3a* (LOC_Os06g06320) *RFT1* (LOC_Os06g06300)	*ZCN8* (GRMZM2G179264) *ZCN12* (GRMZM2G103666)	*SbFT* (Sb10g003940) *SbCN8* (Sb09g025760) *SbCN12* (Sb03g034580) *SbFT12* (Sb06g012260)	*HvFT1* (DQ100327)	*TaFT* (DQ890162)

*Genes on the same row share sequence homology. Each locus corresponds to a unique gene identifier. n.f., not found in public databases; n.p., not present in Arabidopsis.*

Flowering of Arabidopsis is promoted under LD. The circadian clock is responsible for the rhythmic expression of several factors implicated in environmental responses. Among them, the GIGANTEA (GI) and FLAVIN BINDING KELCH REPEAT F-BOX PROTEIN 1 (FKF1) proteins are expressed at the end of the light phase and interact in a light-dependent fashion (Sawa et al., 2007). The resulting complex targets a group of CYCLING DOF FACTORs (CDFs) for proteasome-mediated degradation (Fornara et al., 2009). The *CDFs* encode transcriptional repressors that limit expression of the *CONSTANS* (*CO*) zinc finger transcription factor, a central regulator within the photoperiodic flowering pathway (Putterill et al., 1995). Besides the major GI-FKF1-CDFs module, several additional mechanisms contribute to CO expression at the transcriptional and post-transcriptional level, including regulation by transcription factors (Ito et al., 2012), alternative splicing (Gil et al., 2016), photoreceptors (Valverde et al., 2004; Song et al., 2014), as well as ambient temperature signals (Fernández et al., 2016), hormonal signals (Wang et al., 2016) and post-translational modifications (Sarid-Krebs et al., 2015). However, central to the current model for photoperiodic flowering, the most prominent feature of CO is its light-dependent stability (Valverde et al., 2004; Song et al., 2012). During the night and the morning, CO protein is unstable and quickly degraded (Jang et al., 2008; Song et al., 2014; Lazaro et al., 2015). Consequently, its expression is shaped to be highest under LD, during the light phase. At this time of the diurnal cycle, CO protein, acting in the companion cells of the phloem, can directly promote expression of *FLOWERING LOCUS T* (*FT*), component of the systemic florigenic signal (An et al., 2004; Corbesier et al., 2007; Mathieu et al., 2007).

**FIGURE 1:**
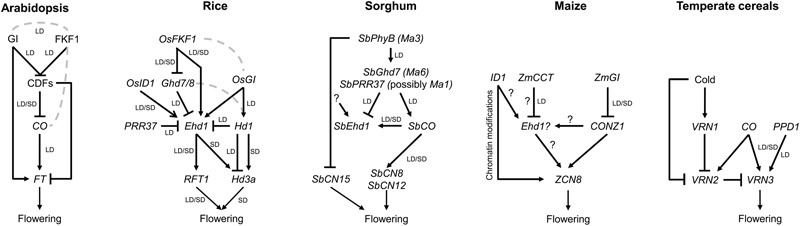
**Simplified genetic modules controlling production of the florigens in leaves.** (Arabidopsis) The photoperiodic flowering pathway can be simplified into a linear cascade comprising the GI and FKF1 protein complex, targeting CDFs transcriptional repressors for proteasome-mediated degradation. Released *CO* transcription leads to CO protein accumulation during the evenings of LD and induction of *FT* expression. Among the CDFs, CDF1 at least can associate directly with *FT* to repress its expression. (Rice) *OsFKF1* and *OsGI* promote flowering via transcriptional regulation of distinct target genes. However, their protein products can also interact. The Ehd1 and Hd1 proteins promote flowering by activating *Hd3a* and *RFT1* expression under SD. However, under LD, Hd1 switches its function to repress *Hd3a* transcription. *Hd3a* transcription is sensitive to induction mediated by *Ehd1* under both LD and SD, to the extent that *ehd1 rft1* double mutants cannot flower. Conversely, transcription of *RFT1* can be activated also under LD, by an unknown mechanism that eventually allows flowering also under unfavorable conditions. (Sorghum) The SbPhyB protein (Ma3) represses flowering by promoting expression of *SbGhd7* (*Ma6*) and *SbPRR37* under LD. The coincidence between *SbPRR37* and *Ma1* is under debate. However, sorghum lines bearing *ma3* recessive mutations can flower early also under SD. The SbCO protein is a constitutive activator of flowering, differently from rice Hd1. *SbPRR37* can promote *SbCO* transcription at dawn. (Maize) A higher degree of polygenic control of flowering has been observed in maize compared to other species. However, homologs of flowering genes have been cloned and some mutants characterized. In this diagram, we speculate about the existence of an *Ehd1*-like function, possibly creating a topology similar to that of other SD species. Major discrete regulators are encoded by *ID1* and *ZmCCT*. (Temperate cereals) Exposure to cold is necessary to reduce *VRN2* levels in leaves. Vernalized plants can respond to LDs that promote expression of *VRN3*/*FT* via *CO* homologs and, most importantly, through PPD1, encoding a CCT-domain protein similar to PRR37. Arrows indicate transcriptional activation; flat-end arrows indicate transcriptional repression. Dashed lines indicate that the protein products can interact. Question marks are speculative, and indicate the possible existence of unknown factors with specific functions on gene expression. LD, long day; SD, short day.

The effects of CO protein on the levels and rhythmicity of *FT* mRNA abundance are mediated by several classes of protein interactors that include transcription factors and transcriptional co-regulators, photoreceptors, histone-like proteins, and ubiquitin ligases (see Brambilla and Fornara, 2016 and references therein). Therefore, the photoperiodic flowering pathway, despite being largely interconnected with other regulatory pathways, can be simplified into a linear molecular cascade, whose major output is the FT protein (**Figure 1**).

## Rewiring Photoperiodic Networks in Rice Modifies Day Length Responses

Rice flowering is accelerated by exposure to SD. Seasonal and diurnal time measurements are mediated by a circadian clock that shares components with that of Arabidopsis, and when mutated results in altered sensitivity to the length of the day (Izawa et al., 2011; Matsubara et al., 2012). Homologs of *GI, FKF1*, the *CDFs, CO*, and *FT* exist in rice and have been partly linked in a cascade that resembles the photoperiodic pathway of Arabidopsis (Shrestha et al., 2014) (**Table 1**). The OsGI and OsFKF1 proteins can interact with each other and with a CDF protein, OsDOF12, similarly to their Arabidopsis homologs (Li et al., 2009; Han et al., 2015). However, mutations in *OsFKF1* delay flowering under any photoperiod tested, whereas *osgi* mutants are late flowering under SD, while having only mild effects under LD (Hayama et al., 2003; Izawa et al., 2011). The phenotypic effects of the two mutations are therefore different. Overexpression of *OsDOF12* increases transcription of *Heading Date 3a* (*Hd3a*), a homolog of *FT*, under LD while having no impact on transcription of *Heading date 1* (*Hd1*), a homolog of *CO*. Thus, the function of *OsDOF12* is opposite to that of Arabidopsis *CDFs*, effectively promoting flowering (Li et al., 2009). It is still unclear whether the interaction between OsGI and OsFKF1 is dependent upon the photoperiod, or if it is necessary for the degradation of OsDOF12 or other DOF proteins. These data indicate that a similar arrangement of regulators exists upstream of *Hd3a*, but that their molecular function or day length-dependency is very different from Arabidopsis. Both the DOF-CO and the GI-FKF1 modules are evolutionarily ancient as indicated by data from the unicellular alga *Chlamydomonas reinhardtii* and the liverwort *Marchantia polymorpha*, where they control phase transition (Kubota et al., 2014; Lucas-Reina et al., 2015). However, evolution has likely re-shaped the function of the dimer several times, readjusting it depending on the species.

Cloning of *Hd1* indicated that it encodes a homolog of *CO* (Yano et al., 2000). However, the Hd1 protein not only promotes flowering under SD but also represses it under LD. Mutations in *Hd1* result in accelerated flowering under LD and have been extensively introgressed in varieties cultivated at high latitudes (Izawa et al., 2002; Hayama et al., 2003; Gao et al., 2014; Gómez-Ariza et al., 2015; Goretti et al., 2017). A second important flowering QTL, *Early Heading Date 1* (*Ehd1*) was later cloned and shown to encode a B-type response regulator (Doi et al., 2004). Ehd1 integrates circadian and light inputs and is required to promote flowering under both LD and SD (Itoh et al., 2010), and to modulate it also in response to abiotic stress, including water deficit (Galbiati et al., 2016; Zhang et al., 2016). Under SD, *Ehd1* induces flowering mainly by promoting *Hd3a* expression, and this function is not shared with dicot species (Zhao et al., 2015). Under LD, expression of *Ehd1* is limited by several repressors that delay flowering, including *Grain Number Plant Height and Heading Date 7* (*Ghd7*), *Hd1*, and *Pseudo Response Regulator 37* (*PRR37*) (Gao et al., 2014; Gómez-Ariza et al., 2015). The Hd1 and Ghd7 proteins interact forming a repressor dimer and at least the Ghd7 protein can directly bind the promoter of *Ehd1* (Nemoto et al., 2016). Thus, genetic and molecular evidences indicate how a conserved inductive cascade has been repurposed and integrated with unique components to create a novel network topology (**Figure 1**).

As with all photoperiodic response networks, the major outputs of the regulatory cascade include *Hd3a* and its paralog *RICE FLOWERING LOCUS T 1* (*RFT1*). Both proteins encode mobile leaf-borne systemic signals, but whereas Hd3a is required only under SD to induce flowering, the RFT1 protein is expressed and can promote flowering under both SD and LD (Komiya et al., 2008, 2009; Zhao et al., 2015). Thus, the facultative response of rice is based on a system comprising two florigens subject to differential regulation. The molecular basis of this differential sensitivity to the photoperiod is still poorly understood.

## Mechanisms of Photoperiodic Flowering in Other Short Day Monocots Including Sorghum and Maize

Sorghum (*Sorghum bicolor*) is a SD plant evolved in Africa, in the Sudan region. Six major QTLs controlling flowering time and termed *Maturity* loci (*Ma1*–*Ma6*) have been detected in sorghum. Almost all QTLs have been identified as photoperiodic flowering regulators and their study is demonstrating the strong homology occurring between the sorghum and rice pathways (Wolabu and Tadege, 2016) (**Table 1**).

Cloning of the *Ma3* locus showed that it encodes *SbPhyB*, a light receptor which can mediate light signaling and flowering repression (Childs et al., 1997). When *SbPhyB* is mutated, sorghum becomes insensitive to the photoperiod and flowers early compared to the wild type both under LD and SD (Yang et al., 2014a). One of the functions of *SbPhyB* is to promote the transcription of *SbPRR37* (possibly *Ma1*) and *SbGhd7* (*Ma6*). These genes encode flowering repressors that limit mRNA expression of downstream targets under LD, including *Ehd1, SbFT*, and *SbZCN8* (collinear orthologs of *Hd3a* and maize *ZCN8*, respectively) (Murphy et al., 2011). The flowering suppressor role of these sorghum genes reflects the function of rice *OsPRR37* and *Ghd7*, indicating that these components are shared among SD cereals. Recent data suggested that the *Ma1* QTL does not correspond to *PRR37*, but rather to an *FT*-like gene, *SbFT12*, that could act as floral suppressor (Cuevas et al., 2016; Wolabu and Tadege, 2016). Additional data will be required to confirm the true identity of the *Ma1* gene.

The regulation of *SbCO* transcription mediated by *SbPRR37* has also been investigated. The data suggest that *SbPRR37* modulates *SbCO* expression at dawn, promoting its transcription under LD, whereas under SD *SbCO* expression seems not to depend upon *SbPRR37* (Murphy et al., 2011). *SbCO* can activate florigen production under both SD and LD conditions through the activation of *SbEhd1, SbCN8*, and *SbCN12* (Yang et al., 2014b). The role of sorghum *SbCO* as constitutive floral activator is therefore different from that of rice *Hd1*, implicating a different regulatory mechanism.

Thirteen different *FT*-like genes have been identified in the sorghum genome, three of which (*SbFT1*/*SbCN15, SbFT8*/*SbCN12*, and *SbFT10*/*SbCN8*) could promote flowering when constitutively expressed in Arabidopsis (Yang et al., 2014b; Wolabu et al., 2016). The transcripts of *SbCN8, SbCN12*, and *SbCN15* peak at dawn but show distinct sensitivities to *SbCO* mutations. Whereas the transcripts of *SbCN8* and *SbCN12* are strongly reduced in the *Sbco* mutant background under LD, *SbCN15* shows only a phase shift, suggesting different regulation by *SbCO* (Yang et al., 2014b). The transcriptional patterns of *SbCN8, SbCN12*, and *SbCN15* under different photoperiods and mutant backgrounds could provide in the future valuable data to understand similarities and differences with the dual florigen system of rice.

Maize (*Zea mays*) was domesticated in central Mexico from Teosinte, which is a SD plant. The first flowering gene cloned in maize was *INDETERMINATE 1* (*ID1*): plants with mutations in this gene delay the floral transition and produce aberrant inflorescences (Colasanti et al., 1998). *ID1* encodes a zinc-finger transcription factor expressed in immature leaves which can activate the floral transition and is not under the control of the circadian clock (Wong and Colasanti, 2007). Although the precise function of *ID1* in the photoperiodic pathway is still unclear, recent analyses demonstrated that *ID1* controls chromatin modifications of loci encoding maize florigens, and that it can regulate flowering through histone methylations (Mascheretti et al., 2015). A rice homolog of *ID1, OsEhd2*, is required to induce *OsEhd1* expression (Matsubara et al., 2008) (**Table 1**). Although a maize *Ehd1* homolog has not yet been found, the high homology between *ID1* and *OsEhd2* could suggest a similar regulatory mechanism, possibly indicating the existence of a ZmEhd1-like protein subject to similar regulation. Indirect evidence supporting this view is that the CCT-domain transcription factor *ZmCCT* shows sequence homology with *OsGhd7*, and encodes a strong LD flowering repressor (**Figure 1**). Mutations in *ZmCCT* cause early flowering and have been artificially selected to expand maize cultivation to higher latitudes (Hung et al., 2012).

Two *GI* homologs are present in maize, *GIGANTEA1* (*GI1*) and *GIGANTEA2* (*GI2*) (Miller et al., 2008). In Arabidopsis and rice, *GI* is under circadian clock control and regulates the expression of several genes important for the floral induction. In maize, *gi1* mutations cause early flowering under LD conditions. Transcriptional analysis of these mutants demonstrated that *GI1* is necessary to repress transcription of *CONZ1* (homolog of *OsHd1*) and *ZCN8* (homolog of *Hd3a*), both of which displayed increased expression in the *gi1* background (Bendix et al., 2013). These data demonstrate that *ZmGI* function is similar to *OsGI* which can repress flowering under LD conditions, a function opposite to that of *AtGI* (Hayama et al., 2003). Whether mutations in *CONZ1* influence flowering is unknown, but the data suggest it to be downstream of *GI1*, and possibly upstream of *ZCN8* as positive regulator of flowering (Miller et al., 2008).

From the analysis of 15 maize *FT*-like genes, *ZCN8* was identified as the strongest candidate for the maize florigen (Meng et al., 2011). *ZCN8* encodes a homolog of *FT* that delays flowering if silenced, and can complement *ft* mutants when expressed in Arabidopsis (Lazakis et al., 2011). The regulation of *ZCN8* is similar to that of another putative maize florigen, *ZCN7*, and is under the control of chromatin modifications governed by *ID1* (Mascheretti et al., 2015). However, whether ZCN7 satisfies the criteria of a florigenic protein is still to be clarified.

## Flowering Mechanisms in Long Day Temperate Cereals

Differently from rice, sorghum, and maize, the temperate cereals wheat (*Triticum* spp.) and barley (*Hordeum vulgare*) were domesticated in the Eastern Mediterranean region, in areas characterized by the alternation of cold and warm seasons. These cereals have evolved mechanisms to prevent flowering when temperatures are low, to protect the meristem from cold damage. Flowering is promoted after exposure to vernalizing conditions, when plants resume growth in the spring. During domestication, some cultivars of these species have lost sensitivity to vernalization and, depending on the response to cold, they could be classified as winter or spring types. Winter-types have an obligate vernalization requirement. Such response is controlled by the *VERNALIZATION* (*VRN*) loci (Ream et al., 2012). *VRN1* is a MADS-box floral promoter homologous to *FRUITFULL* (*FUL*) and *APETALA1* (*AP1*) of Arabidopsis, whereas *VRN2* is a floral repressor sharing sequence similarity to *Ghd7* of rice (**Table 1**). Under low temperatures, the expression of *VRN1* is induced and the protein directly binds to the promoter of *VRN2* to reduce its expression during vernalization (Trevaskis, 2006; Deng et al., 2015). Dominant mutations in *VRN1* or recessive mutations in *VRN2* confer a spring growth habit, and have been exploited by breeders to expand cultivation areas (Yan et al., 2004; Fu et al., 2005; Loukoianov, 2005).

Downregulation of *VRN2* is required to induce *VRN3* expression during the floral transition. VRN3 proteins (designated as TaFT and HvFT in wheat and barley, respectively) are homologs of the Arabidopsis and rice florigens, and move to the apical meristem to promote flowering upon exposure to warm temperatures and LD (Yan et al., 2006; Li and Dubcovsky, 2008). Thus, cold signals coordinate *VRN* expression to activate flowering and long-distance florigenic signaling only when a vernalization requirement has been satisfied.

As soon as *VRN2* levels decrease, exposure to LDs is required to promote flowering. Temperate cereals flower earlier under LDs, whereas exposure to SDs delays flowering. The *PHOTOPERIOD 1* (*Ppd1*) gene has been described as the major factor controlling sensitivity to day length in wheat and barley (Turner et al., 2005; Beales et al., 2007). Mutations in PPD1 delay flowering under LD and reduce *VRN3*/*FT* expression. PPD1 proteins are homologous to PRR37 proteins of rice and sorghum, both of which repress flowering under LD. The functional divergence of PRR37 proteins observed among LD temperate and SD tropical cereals deserves further attention, as it might be at the base of their distinct photoperiodic requirements.

Homologs of *CO* and *Hd1* have been identified in wheat and barley (Campoli and Von Korff, 2014). The *TaHd1-1* gene could complement a rice *hd1* mutant, suggesting functional conservation of protein function in a heterologous system (Nemoto et al., 2003). In barley, studies based on overexpression have provided important clues to the position of *Hd1* homologs in flowering regulatory networks. Overexpression of *HvCO1* and *HvCO2* promoted flowering under both LD and SD, but plants retained sensitivity to the photoperiod, because of independent control of *HvFT1* by *PPD1* (Campoli et al., 2012). Thus, barley flowering depends on two parallel pathways controlling *FT* expression (**Figure 1**). Interestingly, overexpression of *HvCO2* was recently shown to increase expression of *VRN2* under LD and SD in a winter variety (Mulki and von Korff, 2016). Despite such increase of the *VRN2* repressor, overexpression of *HvCO2* could still promote flowering, likely through a *VRN2*-independent pathway. The data might suggest that *HvCO2* mediates a floral repressive function through *VRN2*, to limit *FT* expression. Whether barley orthologs of *Hd1* display dual functions similarly to rice *Hd1* awaits further testing. The use of mutant resources and possibly of edited alleles might help to address this issue.

## Concluding Remarks

The examples discussed above illustrate the flexibility of photoperiodic flowering networks and how adaptation to distinct environments modifies their topology. Major changes include the integration of vernalization modules in some networks and the recruitment of non-shared regulators, such as *Ehd1* and *Ghd7*, in others. A common theme appears to be the requirement for upstream master regulators to control expression of *FT*-like genes, but their number and relative contributions to heading time broadly varies between species. While in Arabidopsis, CO acts as central and primary regulator of *FT*, CO homologs in crops are coupled to parallel pathways largely sharing the workload, and *FT* expression often strongly depends on additional regulators.

Efforts will be needed in the future to isolate all components of the networks in crop species, many of which are still to be cloned. Quantification of transcripts offers a rapid way of determining relationships between genes, but provides only limited information on protein expression or biochemical function. Finally, molecular networks are starting to be built, based on protein–protein or protein–DNA interactions especially in rice. Expanding these efforts toward other crops will prove necessary.

## Author Contributions

FF and VB organized the manuscript and wrote the Arabidopsis and rice sections. MC wrote the maize and sorghum section and prepared **Figure 1**. JG-A wrote the temperate cereals section. FF revised the manuscript.

## Conflict of Interest Statement

The authors declare that the research was conducted in the absence of any commercial or financial relationships that could be construed as a potential conflict of interest.
